# Quantum Light Spectroscopy

**DOI:** 10.1021/acscentsci.6c00757

**Published:** 2026-09-03

**Authors:** Gregory D. Scholes, Quanwei Li, K. Birgitta Whaley, Graham R. Fleming

**Affiliations:** † Department of Chemistry, 168555 Princeton University, Princeton, New Jersey 08544, United States; ‡ Department of Chemistry, University of California, Berkeley, 94720 California, United States; § Kavli Energy Nanoscience Institute (ENSI), University of California, Berkeley, 94720 California, United States; ∥ QB3 Institute, University of California, Berkeley, California 94720, United States; ⊥ Molecular Biophysics and Integrated Bioimaging Division, Lawrence Berkeley National Laboratory, Berkeley, California 94720, United States

## Abstract

Spectroscopy based on individual-photon-level light sources brings new variables to both spectroscopy and imaging. We refer to this approach as quantum-light spectroscopy (QLS). The photon statistics of different types of photon sources and photon entanglements are briefly described. We then illustrate the potential of QLS by means of some current experimental examples including time-resolved spectroscopy performed with single photons and quantum-enhanced optical imaging, showing how all the methods rely on correlations between entangled photons in the pair. We then ask: how might physical chemists exploit QLSs to expose new physical insights? For example, can we address fundamental questions in interactions between light and molecules or materials that are very difficult to address with classical light sources? Does QLS open up techniques to study highly congested spectra with higher combined spectral and temporal resolution than is possible with classical light? The concept of QLS also opens the potential study of fundamental questions such as what initial excited states are produced by individual photon absorption? We also point out the experimental challenges in extending QLS to more complex spectroscopies, such as multidimensional spectroscopies. We conclude with a brief selection of recent theoretical proposals to extend the capabilities of the QLS.

## Graphical abstract

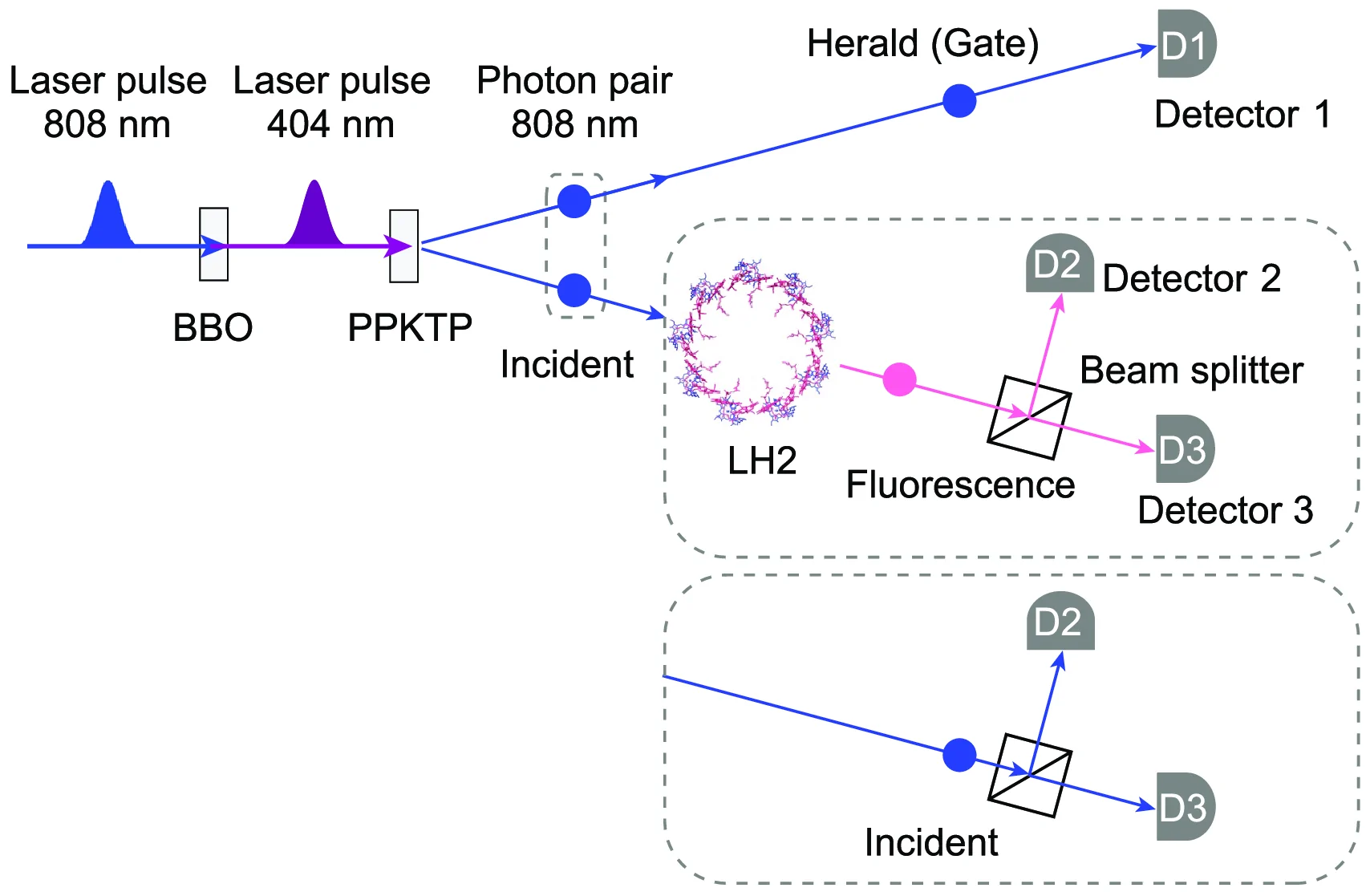

## Introduction

Studies involving light (blackbody radiation, the photoelectric effect, the spectrum of hydrogen) led to the development of quantum mechanics. However, in most applications involving light in chemistry, only the molecule or material was treated quantum mechanically while the light was treated as a classical electromagnetic wave. Over the past 40 years, the notion of using the quantum properties of light to explore and utilize new variables in the study of molecules and materials has emerged. In this Outlook we describe how the quantum properties of light bring new opportunities for molecular spectroscopy and enable enhancement of signal-to-noise ratios. They can also be used to allow signals from awkward-to-detect spectral regions to be recorded in more straightforward spectral regions, and may allow addressing of fundamental questions about the initial state and subsequent dynamics of excited states prepared by optical excitation.

Spectroscopy began in the frequency domain, with developments in the time domain beginning around 75 years ago. These twin approaches have enabled the discovery of many important physical insights. These developments also suggest open questions that appear to be out of reach for current spectroscopic experiments. That realization motivates the exploration of a new class of spectroscopic experiments that is based on individual-photon-level light sources. We refer to this as quantum light spectroscopy (QLS). In this Outlook, we highlight the merits of QLS, which include (i) exciting and probing a system at the quantum level of light, that is, with single or quantum correlated pairs of photons; and/or (ii) exploiting the photon statistics of photon sources/emission in spectroscopic measurements. A discussion of why QLS matters for physical chemists and an outlook on future possibilities in this area are given at the end of the paper. Our main aim in this Outlook is to convey why these attributes of QLS will be valuable for physical chemistry.

## Fundamental Aspects of Light–Matter Interaction

Ever since G. Wilse Robinson first used the phrase ‘exciton spreading’,^1^ debate has continued as to whether natural photosynthetic light-harvesting systems utilize quantum coherence to enhance their remarkable quantum efficiency. The development of two-dimensional electronic spectroscopy and the application to the Fenna Matthews Olson (FMO) bacterial antenna complex^2,3^ in 2005 and 2007 led to a surge of theoretical studies on this question but new types of experiments lagged behind.^4^ Experiments based on femtosecond (laser) pulses are inevitably ambiguous when faced with the question “Can a complex molecular system, excited incoherently, generate coherence through its own internal interactions?” This is because the ultrafast pulses necessarily create coherence (electronic, vibrational or vibronic superpositions) during the sequence of pulses required to carry out the 2D spectral measurements.

Another very different approach to probe the dynamics of molecular systems with optical fields is to analyze photoemission from the excited system, in particular to measure the photon correlations that characterize the quantum behavior of light. In particular, it has long been known that frequency filtering of the second order coherence function i.e., *g* ^(2)^(ω_1_, ω_2_, τ), of fluorescence can reveal coherent couplings in the effective Hamiltonian via observation of time-asymmetric correlation functions.^5–7^ This feature has recently been applied to incoherently excited coupled excitonic systems in a series of pioneering theoretical papers by Olaya-Castro and co-workers,^8–11^ which show that the time and frequency resolved second order coherence function *g* ^(2)^(ω_1_, ω_2_, τ) of emitted fluorescence photons can be asymmetric around τ = 0, with both zero time values and behavior on ps time scales that can reflect the vibronic coherence resulting from near resonant excitonic coupling to underdamped vibrational modes. While other recent theoretical work on *g* ^(2)^(τ) for coupled excitonic systems that has shown that the behavior of *g* ^(2)^(τ) for the fluorescence is still dependent on the nature of the exciting light source, the direct connection of frequency resolved *g* ^(2)^(τ) for fluorescence to vibronic coherence established in ref 6. provides a new experimental tool to access this.

Despite these theoretical advances, measurements of *g* ^(2)^(ω_1_, ω_2_, τ) have not been done with the few ps or fs time resolution necessary to observe the coherence structure, although pioneering experiments by Wiersig et al.^12^ on the transition from thermal emission to lasing of a micropillar laser have demonstrated that *g* ^(2)^(τ) measurements with single ps resolution are possible.

The first experiments using quantum light sources to probe excited state dynamics of chemical systems have recently been carried out, using longer time scales of 100s of ps to ns and incident photon fluxes very similar to that of weak sunlight where bulk studies show that photosynthesis is optimal. While these time scales do not allow probing of excitonic or vibronic coherence during energy transport, they have revealed key features of light harvesting at the single photon level. Here Li et al.^13^ used quantum light sources to explore the simple-sounding question “What is the most probable number of input photons needed to give one fluorescence photon from the acceptor in a donor–acceptor system?” The system used was the LH2 bacterial light harvesting pigment–protein complex and the experimental answer is one photon in for one photon out (see Figure 1), demonstrating a microscopic unit internal quantum efficiency. Li et al. then compared the fluorescence yield, lifetimes, and photon statistics of B850 emission following B800 absorption for single-photon and pseudothermal excitation, to determine whether the photon statistics are modified by the light harvesting dynamics.^14^ The quantum yield and fluorescence lifetime were identical, as were the input and output photon statistics on the ns time scale. Preliminary experiments undertaking *g* ^(2)^(ω_1_, ω_2_, τ) measurements of single molecules have also been carried out on these time scales, employing the semiconducting donor–acceptor polymer IDTBT at room and low (77 K) temperatures.^15^

**Figure 1:**
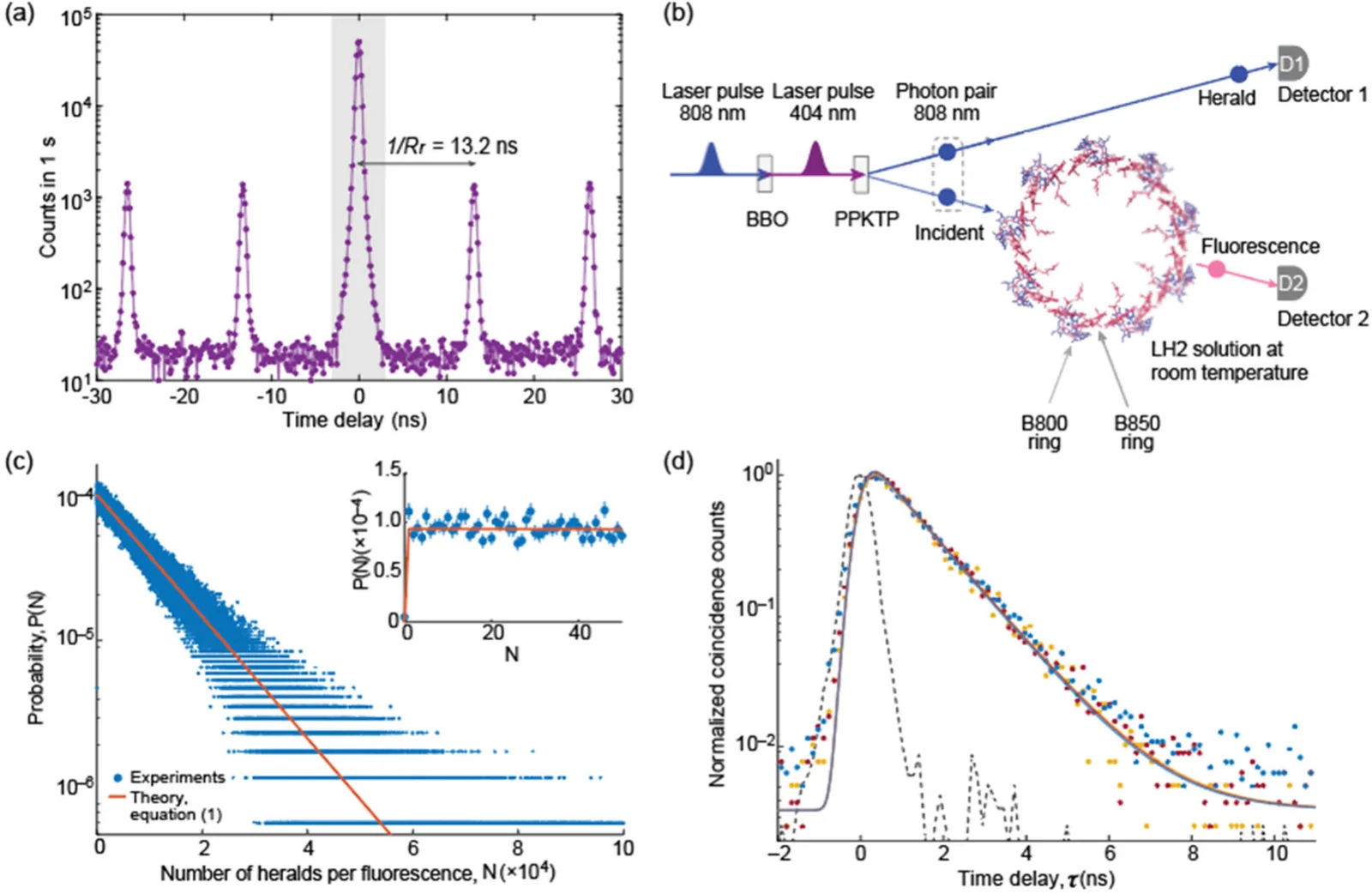
(a) Typical measurement of coincidence counts between herald and incident photons (i.e., between idler photon at Detector 1 and signal photon at Detector 2) as a function of their relative time delay. The main peak at zero-time delay correlates two photons emitted from the same pair, while the smaller side peaks spaced with 1/*R*_*r*_ = 13.2 ns intervals come from the periodic time-shifted random coincidence between photons from different pairs. The gray-shadowed region (6 ns) marks the time window used for calculating the coincidence count rates. (b) Simplified schematic of the time-resolved photon-counting quantum light spectroscopy (PCQLS) for studying single-photon transitions in photosynthetic systems. (c) Experimental, probability distribution of the number of heralds per heralded fluorescent photon, constructed from a total of 1,668,407 heralded fluorescent detections and a total of 17,773,649,622 herald detections, with 5 h integration time. The solid red curves show the prediction of an analytical stochastic model using the measured experimental parameters. (d) Normalized coincidence counts of cross-correlation between heralds and heralded fluorescent photons, plotted as a function of their relative time delay τ with 128 ps bin size. The three sets of colored dots show the measured data at three different incident photon rates represented by the average photon pair number *n*_p_ generated per pump pulse over a 4-fold range. Adapted with permission from Li et al.^13^ Copyright 2023 Authors, licensed under a Creative Commons Attribute 4.0 License CC-BY.

## Absorption of Light

One basic question concerning photosynthesis and other photoinitiated biochemical processes is, what is the time scale for the initial absorption of a photon? Physical chemistry textbooks and even spectroscopy texts in descriptions of the Franck–Condon principle, for example, are rather nonspecific on the question of just how fast the photon absorption process for molecules in condensed phases is, beyond stating that it is very rapid. In many descriptions of spectroscopic studies of molecular dynamics, only the molecular system is treated quantum mechanically, while the light is described as a classical electromagnetic wave. For this situation, the powerful Liouville space description developed by Mukamel^16^ gives a straightforward answer: the time scale of absorption is set by the time scale of dephasing of |e⟩⟨g| or |g⟩⟨e| coherences. This is an accurate approach when there are large numbers of photons in ultrashort laser pulses which typically have 10^10^–10^16^ photons. However, when numbers of photons become small down to a single photon, this approach fails and the quantum nature of light becomes critical.^17^

## Quantum Light

Although we speak colloquially about photon absorption and emission, the basis for traditional spectroscopy and formalisms for light-matter interactions treat light as a classical field. Thus, our light sources are straightforward, and detectors, beam splitters, etc function by classical principles. On the other hand, quantum light needs to be characterized in terms of photon creation and annihilation *operators*. Furthermore, sources, beam splitters, and other optical components function quantum-mechanically. For example, while a beam splitter simply reflects and transmits fractions of an input beam of classical light, a single photon incident on a beam splitter cannot be ‘split’. Instead, it can randomly be transmitted or reflected. Moreover, if the light source is nonclassical, then the output state of the light can be entangled. A quantum-mechanical description of the action of the beam splitter is needed to make these predictions. See Supporting Information.

What does this shift in perspective on the properties of excitation and probe light mean in practice? An important implication is that light sources are not perfect radiators of classical waves. Instead, we should account for how they work at a microscopic basis. For instance, atoms are excited and, with some probability, subsequently emit photons. These emitted photons will thereby be generated according to some kind of statistical distribution over time, which is highlighted by the following examples. Coherent light from a laser source exhibits Poisson statistics where the mean equals the variance of the distribution. Thermal light emitted by a light bulb or the sun shows a super-Poissonian distribution (variance is greater than the mean) because the photon emission is governed by Bose–Einstein statistics.

In contrast to coherent light, where the arrival of photons is random and independent, and thermal light, where photons tend to be bunched together, a single-photon source emits photons that are ordered and antibunched (sub-Poissonian statistics). This special distribution comes about because, after an emitter radiates a photon, it takes some time to be reset by absorption of another photon before a second photon can be emitted. That underlying physical process produces a very regular stream of photons with correlations that are quite distinct from those possible for classical systems.

Photon statistics and correlations embedded in the light source thus come to the forefront when designing and interpreting experiments.

For quantum light sources, it is not only the time correlations between photons that are different from those of classical light sources. In addition, the emitted photons can show nonlocal quantum correlations, specifically, they can be entangled in frequency, time, polarization, or spatial mode. Such entanglement offers the potential for avoiding the time-frequency trade-off imposed by the time-frequency uncertainty principle in conventional spectroscopy. These special aspects of quantum light offer the main resource for photon domain spectroscopy. Indeed, this resource is compelling, because it enables the conceptualization of new kinds of spectroscopic measurements. The potential scope of the QLS program is vast because nonclassical light comes in many forms, with single photons and entangled photon pairs being the most widely studied forms to date. Scheme 1 summarizes some of the new observables and experimental variables that QLS can bring to spectroscopy.

**Scheme 1:**
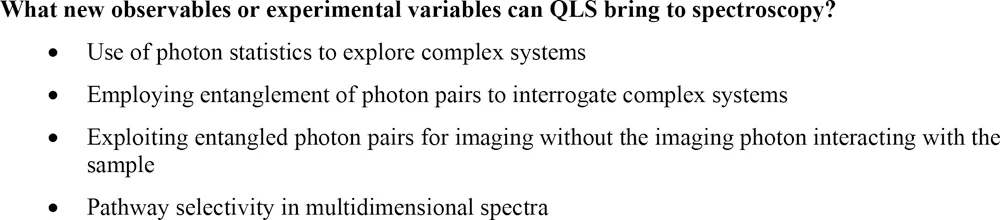


There are many papers containing proposed applications for quantum light spectroscopy, following the influential review of Dorfman, Schlawin, and Mukamel.^18^ Here, we survey current capabilities via recently published experiments to give a snapshot of current methods and achievements. All of these experiments utilize in one way or another the correlations between entangled photons in the pair but do not require both photons to interact with the same molecule or complex. The discussion in the second example below on lowering the flux for two-photon absorption provides the context for this constraint. The number of publications containing experimental data on systems larger than atoms is expanding rapidly, despite the difficulty and complexity of QLS at present. After giving three examples of recent experiments, we close this Outlook with a brief and selective survey of proposed experiments that will likely require substantial developments in creation and detection of entangled photon pairs.

## Example 1: Time-Resolved Spectroscopy with Single Photons

Spontaneous parametric down-conversion (SPDC) provides a source that generates two photons from one photon with frequency equal to the sum of the emitted photons. Type II SPDC produces signal and idler photon pairs with orthogonal polarizations. Entanglement between the photons in a pair produced by SPDC produces both time and frequency correlations between the two photons. The temporal coincidence of these photons (within the entanglement time, which for a biphoton state from SPDC can be understood as the difference in the mean transit time of the two photons across the nonlinear crystal^19^), Figure 1a, can be used to time resolve the fluorescence emission following a single-photon excitation, Figure 1b. In this experiment of Li et al.,^13^ one photon from the signal-idler pair excites the sample, while the other—called the herald—is used as a reference (heralding the time at which the other photon is incident on the sample by a time zero ‘click’ on the herald detector). In Figure 1c the probability of detecting a fluorescence photon when a herald photon is detected is plotted. The exponential tail reflects the differing efficiency of detecting herald and fluorescence photons and shows that the most probable number of heralds for a fluorescent photon to be detected is one. By accumulating a histogram of time differences between a fluorescence photon click on a photodetector that is time correlated with the herald detector, and detection of the corresponding herald photon, the fluorescence emission can be time-resolved, Figure 1d.

The temporal coincidence of these photons can be used to time resolve the fluorescence emission following a single-photon excitation.

In addition to demonstrating the quantum efficiency of light harvesting, this experiment helps resolve the intrinsic kinetics following light absorption by a complex molecular system. By using a single photon, rather than a femtosecond laser pulse, we can find out whether state preparation—for example, by excitation of coherent wavepackets containing on average many photons—influences the short-time dynamics. This has been a perplexing long-standing question, originating from debates over how to understand relationships between measurements of photoluminescent quantum yields and time-resolved fluorescence. Researchers knew from frequency-domain steady-state spectroscopy that population of states redistributes somehow after photoexcitation into a bright state because this is obviously implied by a quantum yield of fluorescence less than unity. However, the dogma that spectroscopy probes *stationary* states (eigenstates of the molecule’s Hamiltonian) painted the field into a corner—stationary states cannot spontaneously undergo dynamics. The early time-resolved experiments were therefore treated with caution.

One concern was whether the kinetics of population transfer, so clearly observed in the time-domain, happened simply because of the laser pulse excitation imprinting coherence on the excitonic dynamics, rather than the pulsed excitation enabling the experimenter to ‘see’ the matter dynamics, intuitively, in the course of time. Such a tension between time-and frequency-resolved viewpoint was echoed by questions as to whether coherent dynamics in two-dimensional electronic spectroscopy happen solely owing to the way state superpositions are prepared using broadband femtosecond laser pulses.^20,21^

In the context of photosynthetic light harvesting, the relevance of laser excitation to study a natural process occurring under excitation by thermal radiation from the sun has been much discussed. The recent experiments with heralded single photons have resolved this issue, allowing spectroscopic probing of the dynamics under photon fluences corresponding to extremely weak sunlight – precisely the conditions under which bulk kinetic studies show that light harvesting is optimized. Further extension of these experiments to employ detectors with fs to ps time resolution^12^ and frequency filtering, combined with theoretical modeling of the dynamics and fluorescence correlations *g* ^(2)^(ω_1_, ω_2_, τ) will now be able to address the question of intrinsic coherence in the excitation dynamics.

In related work, Cerullo and co-workers^22^ and Cushing and co-workers^23^ have exploited the photon correlations in fluorescence emission derived from excitation by ultralow-power continuous laser sources to record fluorescence decays and time-resolved spectra with a few 100 ps time resolution. Experiments with femtosecond resolution of the fluorescence emission using streak cameras^12^ will enable an explicit comparison between the fluorescent photon correlations resulting from excitation by different light sources.

Development of these single- and few-photon methods provides a good outlook for the development of low-power, perhaps even on-chip, devices that could carry out sophisticated spectral/temporal measurements for sensing or diagnostic purposes. Absorption spectroscopy using heralded single photons, a form of frequency-correlated biphoton spectroscopy,^24,25^ has been shown to enable resolution of spectral features below the shot noise limit, allowing high-resolution diagnostic analysis of fragile biological samples such as hemoglobin mixtures in solution with far fewer photon counts than laser spectroscopy.^26^ Time-resolved fluorescence correlation spectroscopy following single- or few-photon excitation could offer similar sensitivity for sensing of dynamical processes in biological systems.

## Example 2: Lowering the Fluence Dependence for Two-Photon Absorption

Two-photon absorption is widely used in excitation microscopy for deep tissue and three-dimensional imaging.^27^ This is in part because the required tight beam focus improves spatial resolution, but also because scattering of the exciting light can easily be discriminated from the fluorescence emission. However, one drawback of this microscopy technique is that a high excitation fluence is needed to ensure that sufficient photons are available for coincident absorption. Researchers have envisioned that this high fluence can be lessened—possibly considerably—by using photon pairs that are time-frequency entangled,^28–31^ as shown in the images of Figure 2. In these images, for similar contrast the quantum two-photon images require ∼5 × 10^6^ lower flux than the classical case.^30^ An obvious ingredient to this reasoning is that these photons are guaranteed to arrive together and can thus facilitate concerted absorption (although they are not guaranteed to be absorbed by the same molecule in a macroscopic sample). A less obvious consideration is that entanglement can provide an enhancement factor over and above the notion of classical coincidence.^19^

**Figure 2:**
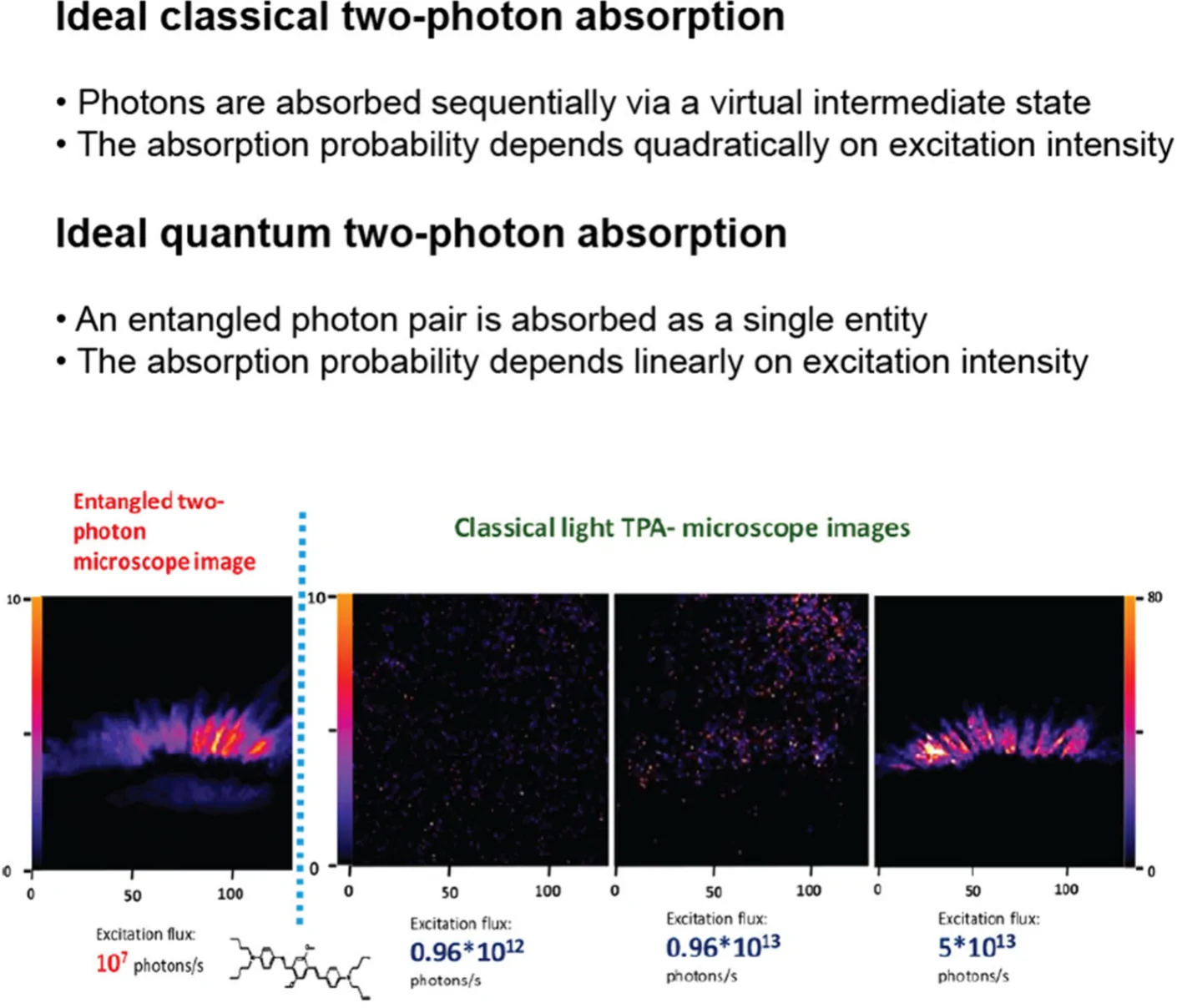
Concepts motivating the development of two-photon absorption using entangled photon pairs. The figure shows a scanning microscope image obtained with entangled photon excitation (left) compared to classical light two-photon excitation at various excitation fluxes for a drop cast film of bis­(styryl)­benzene derivative. Color bar intensity scales are given in numbers of fluorescence photons per pixel. See ref 30. Reproduced with permission from ref 30. Copyright 2020 American Chemical Society.

Entangled photons are produced by the SPDC setup described above. The idea is that regions of the output beams where the signal and idler modes spatially overlap contain not only classically correlated photons but also photons with the stronger quantum correlation of entanglement. Predictions^32,33^ and experiments for atomic systems^34,35^ suggest that these entangled photon pairs allow the two-photon absorption probability to scale linearly with excitation fluence, versus the characteristic, well-known, quadratic dependence. The original demonstration of a linear dependence of a two-photon absorption of a molecular system on the net input rate of entangled photon pairs^36^ generated a great deal of interest and some controversy. Some authors suggested that the linear dependence arose from linear scattering^37^ or hot band absorption.^38^ In the low flux regime, the signal from entangled photon pairs was found to be below the detection threshold with current state-of-the-art detection methods.^39^ More recent work emphasizing the spatial properties of entangled TPA demonstrates that entangled TPA can be observed with careful attention to the spatial and temporal properties of the photon-pair source.^40^ In parallel, an improved understanding of entangled TPA cross sections has allowed comparison of how the ratio of entangled and classical TPA cross sections depends on molecular electronic structure.^41^

Exploit entangled photon pairs for imaging without the imaging photon interacting with the sample.

Although debate on contributions of one-photon processes to entangled TPA continues,^42^ the differing spectral trends of entangled and classical TPA cross sections^43^ raise the intriguing possibility of differing photochemical outcomes with the two sources. Much remains to be quantified and reliably observed and predicted in entangled two-photon absorption, but the remarkable potential for imaging, sensing, and quantum information science suggests this area will remain a vibrant aspect of modern spectroscopy.^44,45^

For the present purposes, the examples above elegantly highlight the potential for improving the sensitivity of spectroscopy by leveraging quantum correlations.

## Example 3: Quantum-Enhanced Optical Imaging

When the quantum nature of light is taken into account, then new imaging methods become possible. For instance, quantum light images that have been recorded with noise levels below the shot noise, surpassing the resolution limit, or working at very different wavelengths than that transmitted through the sample. The correlations between photon pairs generated by SPDC persist even when their wavelengths are widely separated, for example, infrared for one photon and visible for the other. This has enabled infrared microscopy detected by visible photons based on the phenomenon of induced coherence. Krivitsky and co-workers have used this method to build an IR hyperspectral microscope with off-the-shelf components built for visible light.^46^

When the quantum nature of light is taken into account, new imaging methods become possible.

The Heisenberg uncertainty principle limits the precision with which the intensity and phase of an optical field can be known simultaneously. However, by increasing the noise in the phase, the noise in the intensity can be reduced – a process known as squeezing. In an imaging experiment, where the spatial intensity is the observed quantity, squeezing enhances the signal-to-noise ratio. For example, Bowen and co-workers have used amplitude squeezed light to enhance optical tracking of lipid particles, surpassing the quantum noise limit by 42%^47^ and more recently employed squeezed Stokes frequency light to improve the signal-to-noise ratio in a Raman microscopy of intact yeast cells by 35%^48^ while Scully and co-workers have used squeezed light to minimize sample damage in stimulated Brillouin scattering studies of viscoelastic properties of cancer cells and brain tissues.^49^ A review of advances in squeezed light with application to spectroscopy and microscopy of biological samples can be found in ref 50.

A second approach to the use of quantum light in microscopy is to exploit the entangled two-photon absorption to record images at much lower flux than classical TPA and minimize sample damage. A reduction of 10^6^ in intensity compared to that required for classical TPA microscopy has been reported and applied to breast cancer cells, brain tissue, and the plant root-rhizobacteria system.^51–53^

Another quantum light tool that has been used to make high-precision measurements of distance, with potential application to biological samples, is Hong-Ou-Mandel (HOM) interferometry. This relies on the interference of indistinguishable photons, with any relative path length delay causing detectable changes in the interference signal (see the Supporting Information). This allows high-precision measurement of time delays as small as attoseconds (10^–18^ s), which have been used to measure distances of 1 nm or less in optically transparent samples relevant to cell biology.^54^

Excellent reviews of this developing field of quantum light for imaging and related nanometer-scale mapping of biological and materials samples are given by Defienne et al.^55^ and Tsao et al.^56^

## Looking Ahead: Single-Photon-Scale Excitation and Subsequent Dynamics

The availability of increasingly sophisticated and robust sources of single photons and entangled photon pairs, accompanied by similar advances in single-photon detector technology, raises the possibility of experimentally probing fundamental issues in the interaction of light with molecular systems. Molecular systems are generally open quantum systems prepared in bulk samples, typically in the condensed phase, where there are significant intermolecular interactions. This means that, in practice, an isolated molecular eigenstate cannot be populated by single-photon absorption. Taken together with the fact that excited-state potentials and environment degrees of freedom have equilibrium geometries different from those of the ground-state system, it is to be expected that any photoexcitation conditions will initiate excited-state dynamics and that these dynamics may involve both electronic and nuclear degrees of freedom.

Record signals with higher sensitivity then can be achieved by classical light sources, and at wavelengths more convenient to detect than the wavelength used for probing/imaging.

This Outlook motivates two basic questions for single-photon and entangled-photon excitation of molecular systems. First, what initial excited states are produced by the individual photon absorption? What is the time scale for this? Does two-photon absorption by entangled pairs, even if very low in probability, create different initial excited states than mixed states of photon pairs? Second, how does the absorption of an individual photon influence the subsequent excited-state dynamics in a complex molecular system? This relates to the oft-discussed question of whether thermal and coherent light sources can both induce coherent dynamics. At the single-photon level, this question is in sense moot, since a coherent state with unit average photon number is dominated by a single photon.

It is useful to distinguish questions about mechanism and ‘how’ coherence arises from questions about the functional relevance of this in biochemical systems, whether the coherence is generated by photoexcitation,^57^ or by the system itself.^58,59^ In both cases, however, close interplay between experiment and theory is required. Theoretical studies for light harvesting systems have shown that on average single photon excitation generates coherently coupled exciton–phonon dynamics that differ from dynamics generated by laser excitation only in the absence of coherence between the ground and excited state.^60^ Whereas, quantum trajectories allow the spatiotemporal evolution in the excited system following absorption of individual photons to be monitored by conditioning on fluorescence emission^61^ as well as on changes in vibrational excitation of strongly coupled phonon modes, if it is possible to monitor the latter. Averaging over such single photon trajectories recovers the near unit quantum efficiency measured for single photons by Li et al.^13^ Simulating the dynamics of individual single photon absorption and subsequent excited state dynamics requires a theory tool box including both quantum optics and open quantum systems tools, in addition to the now relatively mature simulation methodologies for excitons and their couplings to vibrations developed in the chemical physics and ultrafast spectroscopy communities.

However, we should not underestimate the experimental challenges in realizing experiments probing dynamics following excitation by single photons. A desirable next experiment following that of Li et al.^13^ for light harvesting systems would be to perform a pump–probe experiment using one of the two entangled photons from the SPDC as a pump photon and time delaying the other photon to use it as the probe photon. Here the quantum light would be used as a direct probe of the excited state dynamics. But the low absorption probability of chlorophylls and related molecular complexes (∼10^–6^),^17^ combined with the low efficiency of production of photon pairs by the SPDC (∼10^6^ pairs/s) means that the likelihood of one light harvesting complex absorbing both pump and probe photons is too low to realistically perform the experiment. A future route to feasibility of this direct method for exploring dynamics following single photon excitation could be to put the sample into a cavity to enhance the absorption probability, with the caveat that the system may then be significantly obscured by polaritonic effects. The advent of experiments measuring frequency selective and time-resolved *g* ^(2)^(ω_1_, ω_2_, τ) using different excitation sources, coupled with corresponding detailed realistic molecular level calculations will provide an alternative novel route to address this key issue for understanding the mechanism of important photoinitiated biomolecular processes such as light harvesting.

To address the dynamics and functional significance of coherence raises many important open questions that take us from the present status of observing coherence to elucidating its possible functional role, or how much it changes function. One approach to this is to assess, for some dynamics process and measurement, whether a chosen quantum resource (e.g., coherence) can have a functional influence, and by how much. These kinds of questions require a framework that defines the free baseline for a chosen quantum feature in a model-independent manner. Quantum resource theories, such as one recently developed for light-induced dynamics,^62^ provide one appropriate framework. Here one specifies which states are ‘free’, meaning they do not possess the resource of interest, and considers that all other states are useful resources. In spectroscopy, nonfree or resourceful initial states may arise, for instance, from ultrafast excitation preparing coherent superpositions in the site basis or from initially delocalized excitons spanning donor and acceptor sites.^63^ Thus, the bright states produced by single-photon absorption are resourceful states, consistent with the conclusions of refs 14 and 60.

## Outlook: What Can Physical Chemists Gain from Quantum Light Spectroscopy?

What are some future possibilities in quantum light spectroscopy for advancing physical chemistry? In Scheme 2, we begin by listing some advantages that QLS might offer compared to more standard spectroscopies.

**Scheme 2:**
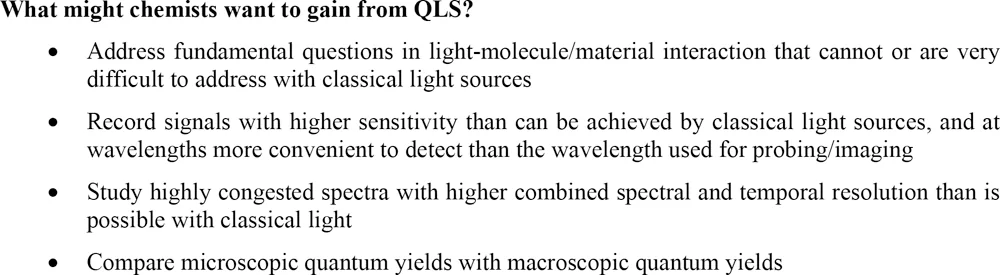


The theoretical literature contains a multitude of proposed novel experiments involving entangled photon pairs and photon statistics. Because of the comparatively low rates of entangled pair production in down-conversion sources and the lack of widespread access to high-quantum-efficiency photon-counting, timing, and array detectors, many of the proposed experiments are extremely challenging at present. As the potential impacts of quantum light spectroscopy become increasingly apparent, the more widespread use of single-photon sources and single-photon detectors will enable a wide range of new types of spectroscopy. Most of the proposed experiments involve two or more entangled photons interacting with the same molecule or complex, suggesting that single-molecule/complex methods will be necessary. Optical cavities may also help, as mentioned above. Below we summarize three enticing possibilities:

1. Breaking the Fourier limit on time and frequency resolution.^63^ Because the time (typically emission time in SPDC) and frequency distribution of entangled photon pairs are not conjugate variables, should both photons be able to interact with the same molecule in a pump–probe configuration, it would become possible to resolve energy levels and dynamics in multilevel systems characterized by rapid interlevel dynamics. However, it has been shown recently that suitably shaped classical laser pulses can also enable sub-Fourier limit spectroscopy and could provide the same information.^64^ Of interest now is to identify higher-order experiments with entangled photon pairs that also probe internal matter dynamics that cannot be emulated with tailored-shaped classical pulses.

2. Signals recorded at more convenient wavelengths. Infrared spectra are vital resources for analysis of materials, medical diagnostics, and environmental sensing. The detection equipment for infrared light is expensive and less sensitive than that used for visible light. By generating both infrared and visible photons by SPDC, it has been shown that the absorption coefficient and refractive index of a medium in the infrared can be recorded by the intensity interference pattern of the visible photon.^65^ In a similar vein, mid-infrared spectra have been recorded by an upconversion of mid-IR photons generated by SPDC.^66^

3. Multidimensional optical spectroscopy has revolutionized the study of both structure and dynamics, revealing pathways and mechanisms of relaxation in complex systems and stimulating considerable developments in the theory of condensed phase chemical dynamics.^4,67^ If 2D electronic spectroscopy can be accomplished with entangled pairs of photons, a number of new insights have been suggested to be available. As multidimensional spectroscopy is applied to ever more complex, multicomponent systems, methods that might allow specific simplification by selectively enhancing or suppressing specific peaks will be very valuable.^68,69^ In addition, addressing two-exciton states could provide insight into exciton fusion, exciton–exciton annihilation, and other multiexciton phenomena.^70^ The difficulty of implementing more complex optical spectroscopies with the low flux sources typically possible for single or entangled photon sources has led to the consideration of intense sources generated by high-gain down conversion,^71^ but as yet such methods have not been applied experimentally to multidimensional spectroscopy.

## Supplementary Material



## Notes

Classical and quantum descriptions of a beam splitter (PDF)

## +

College of Chemistry and Molecular Engineering, Peking University, Beijing 100871, China
