# Supplementary material for: Quantum Light Spectroscopy
Source: ACS Cent Sci. 2026 Sep 3;12(9):1295–304. doi: 10.1021/acscentsci.6c00757 (PMC13621430; doi:10.1021/acscentsci.6c00757)
Supplement: Supplementary file 1 [file oc6c00757_si_001.pdf]

Supporting Information for Quantum Light Spectroscopy

Authors: Gregory D. Scholes[1*], Quanwei Li[2,3+], K. Birgitta Whaley[2,3*], and Graham R. Fleming[2,3,4*]

[1]Department of Chemistry, Princeton University, Princeton, New Jersey 08544 USA

[2]Department of Chemistry, University of California, Berkeley, California 94720 USA

[3]Kavli Energy Nanoscience Institute (ENSI), University of California, Berkeley, California 94720 USA

[4]QB3 Institute, University of California, Berkeley, California 94720, Molecular Biophysics and Integrated Bioimaging Division, Lawrence Berkeley National Laboratory, Berkeley, California 94720 USA

+ Present address: College of Chemistry and Molecular Engineering, Peking University, Beijing 100871, China

The Beam Splitter

A classical beam splitter works intuitively. An input beam with complex field amplitude E_1 enters the beam splitter from the left (call this port 1). The reflected part of the input beam gives field amplitude E_2, that emerges from port 2, while the transmitted fraction, E_3, emerges to the right, from port 3. We require that the intensity of the incident beam is distributed among the output beams, so that $|E_1|^2 = |E_2|^2 + |E_3|^2$.

To translate this model to the quantum case (see, e.g., Chapter 6 of Ref. 72[1]), one could try to simply replace each E_i by a corresponding annihilation operator a_i. However, this does not work out because one finds that the field operators do not both predict the correct fractions of transmitted and reflected light *and* obey the appropriate commutation relations. It turns out that the correct field operators must, surprisingly, include *both* possible input ports. That is, one needs to account for the vacuum field input into the top of the beam splitter (call this input port 0) even though light is only input into input port 1 in practice. Using scalars r and t to indicate the fraction of the beam entering port 1 that is reflected and transmitted, and similarly r' and t' for the fictional beam entering port 0, one obtains the following relationship between annihilation operators of the input and output ports:

$$\begin{pmatrix} a_2 \\ a_3 \end{pmatrix} = \begin{pmatrix} t' & r \\ r' & t \end{pmatrix} \begin{pmatrix} a_0 \\ a_1 \end{pmatrix} = U^\dagger \begin{pmatrix} a_0 \\ a_1 \end{pmatrix} U, \tag{1}$$

where $|r'| = |r|$, $|t'| = |t|$, $|r|^2 + |t|^2 = 1$, $r^*t' + r't^* = 0$, and $r^*t + r't'^* = 0$, and U is a unitary operator. Here * means complex conjugate and the dagger means Hermitian conjugate.

Now one can predict how a beam splitter acts on single incident photons. Start with the fact that the vacuum transforms as $|0\rangle_0|0\rangle_1 \rightarrow |0\rangle_2|0\rangle_3$. Now write the input of a single photon into port 1 in terms of a creation operator: $a^+_1|0\rangle_0|0\rangle_1$. A typical 50:50 beam splitter shifts the phase of one beam relative to the other. For simplicity, without loss of generality, one can assume that the reflected beam experiences as $\pi/2$ phase shift. In this situation

$$\begin{aligned} a_2 &= \frac{1}{\sqrt{2}}(a_0 + i a_1) \\ a_3 &= \frac{1}{\sqrt{2}}(i a_0 + a_1) \end{aligned}, \tag{2}$$

yielding $a^+_1 = (ia^+_2 + a^+_3)/\sqrt{2}$ and thus the beam splitter acts as follows:

$$|0\rangle_0|1\rangle_1 \rightarrow \frac{1}{\sqrt{2}}(ia_2^\dagger + a_3^\dagger)|0\rangle_2|0\rangle_3) = \frac{1}{\sqrt{2}}(i|1\rangle_2|0\rangle_3 + |0\rangle_2|1\rangle_3), \tag{3}$$

showing that if the photon emerges from port 2, nothing comes from port 3, and *vice versa*. The photon will randomly be either transmitted or reflected—as expected. More profoundly, the output state is an entangled state of photon number and position.

Using this same formalism, it can be shown that a classical-like state of light, such as a coherent state, does not generate entangled states when passed through a beam splitter. Moreover, two indistinguishable single photons (e.g. photons generated by an ideal quantum light source), each incident in a different input port, will always emerge together from the *same* output port. In this case, we have

$$|1\rangle_0|1\rangle_1 \rightarrow \frac{1}{2}(a_2^\dagger + ia_3^\dagger)(ia_2^\dagger + a_3^\dagger)|0\rangle_2|0\rangle_3$$
$$= \frac{i}{\sqrt{2}}(|2\rangle_2|0\rangle_3 + |0\rangle_2|2\rangle_3) \quad (4)$$

This is an example of two-photon interference, where a dip in the coincidence counts for photons emerging from the beam splitter is found, signaling that the photons are indistinguishable in all respects, including that they enter the input ports at the same time. It is known as the Hong-Ou-Mandel effect after its developers[2].

References

(1) Gerry, C.; Knight, P. *Introductory Quantum Optics*.

(2) Hong, C. K.; Ou, Z. Y.; Mandel, L. Measurement of Subpicosecond Time Intervals between Two Photons by Interference. *Phys. Rev. Lett.* **1987**, *59* (18), 2044–2046. https://doi.org/10.1103/PhysRevLett.59.2044.
